# The balance of the sexes

**DOI:** 10.7554/eLife.83254

**Published:** 2022-10-07

**Authors:** Ralf HJM Kurvers, Lysanne Snijders

**Affiliations:** 1 Center for Adaptive Rationality, Max Planck Institute for Human Development Berlin Germany; 2 Department of Biology and Ecology of Fishes, Leibniz Institute of Freshwater Ecology and Inland Fisheries Berlin Germany; 3 Behavioural Ecology Group, Wageningen University Wageningen Netherlands

**Keywords:** cooperation, competition, sexual selection, sexual conflict, bird, ostrich, Other

## Abstract

A large-scale experiment demonstrates sex differences in cooperation and competition that can explain group size variation in ostriches.

**Related research article** Melgar J, Schou MF, Bonato M, Brand Z, Engelbrecht A, Cloete SWP, Cornwallis CK. 2022. Experimental evidence that group size generates divergent benefits of cooperative breeding for male and female ostriches. *eLife* **11**:e77170. doi: 10.7554/eLife.77170.

Two is company, three is a crowd – but not for everyone. Animal group sizes vary considerably both across and within species. Explaining why this variation exists is a central question in sociobiology (Clutton-Brock, 2021; Rubenstein and Abbot, 2017). Social companions provide real perks; for instance, cooperative breeders share the costs of parental care. But individuals generally have their own interests at heart, and so the risk of competition is never far away. This is especially true for animals that are cooperative breeders but form breeding groups in which members are not related, such as ostriches (*Struthio camelus*). In such groups, individuals only benefit from fitness through their own offspring, so the size of these groups is the result of a balancing act between two major social forces: cooperation and competition.

In the wild, the sizes of ostrich breeding groups living under similar ecological conditions vary widely, from single pairs to groups of up to 20 individuals. Moreover, these groups often differ in their ratio of males to females. This heterogeneity in group composition may be a clue to understanding variations in breeding group size that are independent of ecology: the benefits of cooperation and costs of competition are not always shared equally between the members of a group (Jolles et al., 2020; Ward and Webster, 2016), leading to different social preferences. In other words, the optimal group size and sex ratio for reproductive success may be different for male and female ostriches. Now, in eLife, Charlie Cornwallis and colleagues from Lund University, the University of Stellenbosch and the Directorate of Animal Sciences – with Julian Melgar as first author – report on how manipulating both group size and sex composition of ostrich breeding groups can be used to test this prediction (Melgar et al., 2022).

Melgar et al. took ostriches from a breeding farm in South Africa and formed breeding groups of different sizes and compositions in separate enclosures. Each group contained 1, 3, 4, or 6 females, and 1 or 3 males, comparable to the natural variation in groups in the area. Melgar et al. also manipulated one of the likely benefits of cooperative breeding in ostriches: shared incubation. By studying optimal group size – defined as the group size at which an individual produces the most chicks – and how it changes with the presence or absence of shared incubation, it was possible to test whether the benefits of cooperative incubation differed between males and females.

Each of the breeding groups underwent two conditions. In one condition, recently laid eggs were removed and placed in incubators for hatching; in the other, the eggs were left for the ostriches to incubate. For males, both treatments had the same result: the number of chicks per male always increased with the number of females in the group, and always decreased when the number of males increased (Figure 1A). These results indicate that, for males, optimal group size for reproductive success depends more on the costs of competition with other males than on the benefits of shared incubation. For females, however, the results looked different. When the eggs were removed, the size and composition of the group did not have a clear effect on female reproductive success. However, when the eggs were incubated by the ostriches, female reproductive success depended on the number of males and other females in the group. This result indicates that incubation sharing is a relevant factor explaining optimal group size for females.

**Figure 1.**
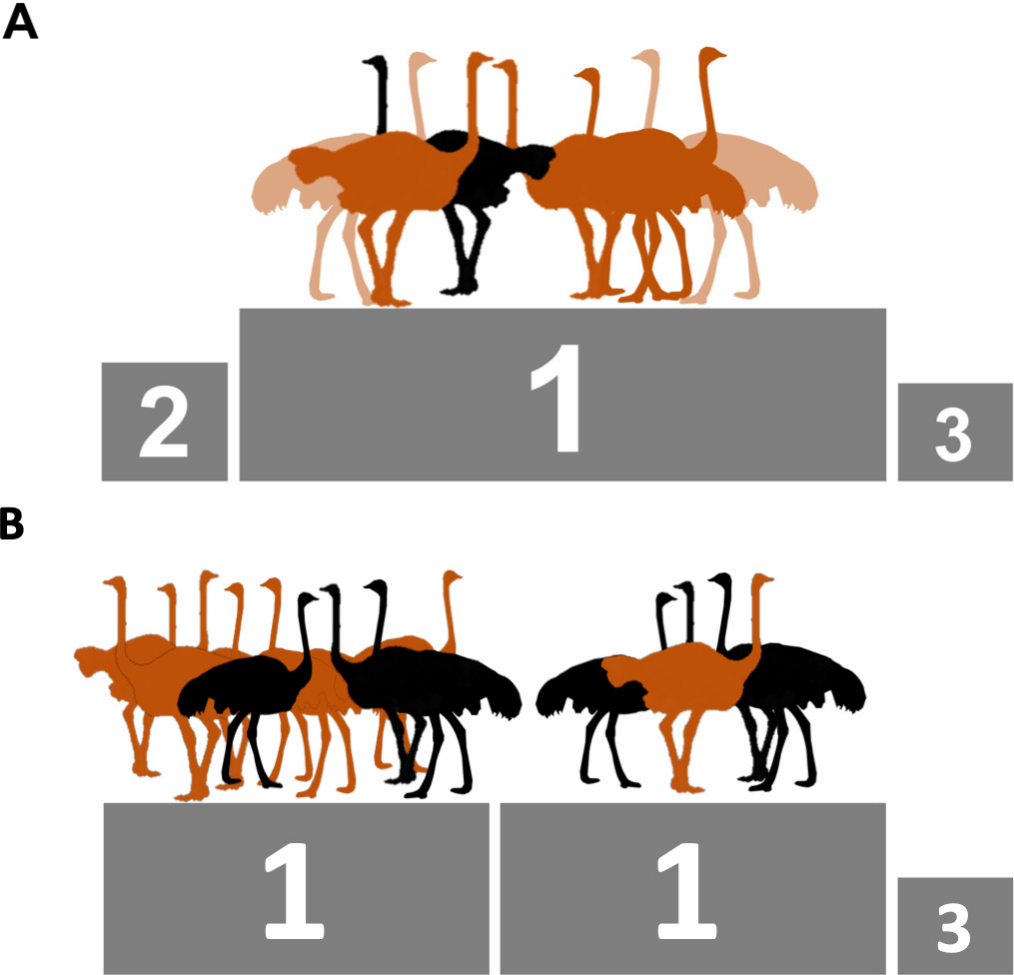
Ostrich group size and composition which maximizes reproductive success during incubation. (**A**) In groups with a single male, both males (black) and females (brown or tan) do better (indicated by a 1 on the podium) with more females around. (**B**) In groups with three males, the males do better with many (six) females around (left). However, when there are three males, females do best when they are either one of many (six) females in the group (left) or when they are the only female (right); meaning that these two combinations share the number 1 position.

Interestingly, males had a clear optimal group size: more females were always better for reproductive success. In contrast, for females more females were only the best solution if there was a single male in the group (Figure 1A). In groups with three males, on the other hand, females did better when they were either the only female, or one of six (Figure 1B). The low performance of females in intermediate groups could be partly explained by sexual conflict. In intermediate groups with three males, females were interrupted more often while they were incubating, leading to eggs being broken following sexual harassments by ‘slacking’ males (i.e. males that spent less time incubating than females). For females, reproductive success is thus maximized across multiple group sizes and compositions due to the opportunity for females to share incubation costs and minimize sexual harassment.

Melgar et al. also measured how much time ostriches spent incubating eggs. Unsurprisingly, they found that the larger the group size, the larger the proportion of time eggs were incubated, leading to greater hatching success. More importantly, individuals could benefit from this increase in total incubation time without having to increase their own time spent on the task – demonstrating that, for ostriches, shared incubation is an important benefit of breeding in larger groups.

Natural variation in the social organization of cooperative breeders is abundant but causal demonstrations of mechanisms to explain this variation are rare (Balshine et al., 2001; Clutton-Brock, 2021; Rubenstein and Abbot, 2017; Shen et al., 2017). Using ostriches, a key model system for cooperative breeding (Bertram, 2014), Melgar et al. have taken a crucial step in understanding variation in the size of breeding groups. By experimentally manipulating group size and composition as well as a key benefit of cooperative breeding, they have revealed how sex differences in cooperation and competition can play an important role in explaining natural breeding group variation.

Of course, some questions remain to be answered. The optimal group sizes observed in the enclosures were generally larger than those observed in the wild, which could be explained by additional selection pressures that were excluded from these experiments. Future studies could improve on the design by allowing for natural variation in food availability and the presence of nest predators. Larger groups are more conspicuous and may therefore suffer more nest predation and suffer more under competition for food, creating a shift to smaller optimal group sizes (Krause and Ruxton, 2002). In addition, sex is unlikely the only trait to generate differences in optimal group size between individuals. Traits such as age, dominance, and personality are also thought to be significant (Jolles et al., 2020). Identifying the traits that causally influence optimal group size in cooperative breeders through carefully-designed experiments is an intriguing direction for future studies.
